# WHO CAN BENEFIT FROM HOME-BASED MEDICAL CARE?

**DOI:** 10.1093/geroni/igac059.1627

**Published:** 2022-12-20

**Authors:** Katherine Ornstein, Bruce Leff, Jennifer Reckrey, Evan Bollens-Lund, Margaret Salinger, Yihan Wang, Christine Ritchie

**Affiliations:** Johns Hopkins University School of Medicine, Baltimore, Maryland, United States; Johns Hopkins University School of Medicine, Baltimore, Maryland, United States; Icahn School of Medicine at Mount Sinai, New York, New York, United States; Icahn School of Medicine at Mount Sinai, New York, New York, United States; Harvard Medical School/Massachusetts General Hospital, Boston, Massachusetts, United States; Icahn School of Medicine at Mount Sinai, New York, New York, United States; Massachusetts General Hospital, Boston, Massachusetts, United States

## Abstract

Leaving the home to access medical care may result in undue burden for patients with dementia and other serious illnesses and their caregivers. While home-based medical care (HBMC) may be beneficial for many older adults, it is not clear how to best identify individuals who could benefit from such services. Using the 2015 NHATS linked to Medicare claims we estimated prevalence across multiple overlapping subtypes: Individuals who have moderate/severe dementia; are homebound; have serious illness; are frail; rely on assistive devices; have high caregiving needs; those with minimal primary care and high ED use; and those who met previously established criteria for Independence at home. Using these criteria, more than half of community-dwelling older adults could benefit from HBMC and more than 25% meet multiple criteria. Medicare and other payers can benefit from targeted identification of patients who could benefit from HBMC.

